# Supplementary material for: Factors associated with uptake of breast and cervical cancer screening among Nepalese women: Evidence from Nepal Demographic and Health Survey 2022
Source: PLOS Glob Public Health. 2024 Mar 11;4(3):e0002971. doi: 10.1371/journal.pgph.0002971 (PMC10927089; doi:10.1371/journal.pgph.0002971)
Supplement: S1 File — (DOCX) [file pgph.0002971.s001.docx]

# **S1 File**

| **Independent variables** | **Categories** | **Definition** |
| --- | --- | --- |
| Ecological belt | Mountains, Hills, and Terai | Ecological classification of Nepal into mountain, hill and terai |
| Place of residence | Urban, Rural | The classification of place of residence into rural and urban is based on the type of municipalities in which the women are residing. |
| Province | Koshi, Madhesh, Bagmati, Gandaki, Lumbini, Karnali, Sudurpaschim | Administrative classification of Nepal into 7 provinces. |
| Ethnicity | Brahmin or Chhetri, Dalit, Janajati, Madhesi, Other | This classification is based on the classification made in DHS report of Nepal |
| Religion | Hindu, Non-Hindu | Non-Hindu include Christian, Islam, Kirat and other religions |
| Marital status | Unmarried, Married or living together, Divorced, or not living together | This classification is based on the marital status of the woman |
| Wealth quintile | Poorest, Poorer, Middle, Richer, Richest | Wealth quintile classification is based on the wealth index calculated |
| Education | No education, Basic, Secondary, Higher | Basic education includes education from grade 1 to 8; Secondary education include education from grade 9 to 12; Higher education include more than secondary (grade 13 and above). No education refers to have no formal education. |
| Occupation | Not working, agriculture, professional or technical or manager or clerical, sales and service, skilled or unskilled labor, others | This classification is based on the classification made in DHS report of Nepal |
| Covered by health insurance | Covered, Not covered | The woman is considered to have covered by health insurance if she reported that she is enrolled in the health insurance program. |
| Media exposure | Present, Not present | The women were considered to have mass media exposure if the women reported reading newspapers, watching television, or listening to the radio at least once a week. |
| Heard about cervical and breast cancer | Yes, No | The women were considered to have heard about cervical/breast cancer if they reported to have heard about breast or cervical cancer |
| Heard about cervical and breast cancer screening | Yes, No | The women were considered to have heard about cervical/breast cancer screening if they reported they have listened about breast or cervical cancer screening |
| Parity | Nullipara, Primipara, Multipara | A woman who has never carried a pregnancy beyond 20 weeks is nulliparous. A woman who has given birth once is primipara. A woman who has given birth two, three, or four times is multipara. |
| Distance to health facility | Big problem, Not a big problem | This classification is based on how woman perceived and report about distance to health facility. |
