# Supplementary material for: Factors associated with uptake of breast and cervical cancer screening among Nepalese women: Evidence from Nepal Demographic and Health Survey 2022
Source: PLOS Glob Public Health. 2024 Mar 11;4(3):e0002971. doi: 10.1371/journal.pgph.0002971 (PMC10927089; doi:10.1371/journal.pgph.0002971)
Supplement: Attachment — Submitted filename: Resposne to reviewers.docx [file pgph.0002971.s002.docx]

Dear editor and reviewers,

We would like to thank all the reviewers and editor for providing their important time and effort in reviewing our manuscript. We have addressed all comments and answer to all comments are listed below:

1. **Around one-third of women respondents being less than 20 years of age, has HPV vaccination been included in the questionnaire?**

Response: No HPV vaccination is not included in the questionnaire of Demographic and Health survey, so we have not included it in this paper.

2. **Has the authors explored the reason behind Dalit women having better attitude towards screening uptake than the socially considered upper class of Brahmin/Chettri women?**

Response: Nepal consists of people from various ethnicities, with Brahmin/Chhetri being regarded as a high caste or advantaged group, and Janajati and Dalit as a lower or disadvantaged group. Among disadvantaged groups, such as Dalits or Janajatis, women are more empowered and capable of making their own decisions. These women, despite their poor financial status, are relatively easy to convince to adopt health-seeking behavior. This may explain the higher likelihood of BCS uptake among Dalit women compared to Brahmin women.

3. **My main concern is with the age group of women who are being asked about screening, given that cervical screening is not recommended to commence before age 30 in most settings and breast screening typically at 40-50 years. I think the analysis and interpretation would really benefit from considering this, particularly in relation to reported cancer screening uptake.**

Response: We have incorporated your suggestions to our analysis.

4. **Are you also able to provide more information about who provides screening services in Nepal and what their capacity is (i.e. if more women want to screen is it possible? Where are these services located? Is it affordable to most people? Is awareness and acceptance the major barrier to screening or is the availability of and access to services also a major issue?)**

Response: Thank you for your feedback. We have included all possible information as per your suggestion.

5. **Line one, Fix wording – insert ‘a’ before major and replace ‘the’ with ‘a’ leading. ie Cancers are a major… and a leading cause of death.**

Response: corrected

6. **Last sentence – 90% of which new cases and deaths – from cervical cancer or something else? Specify.**

Response: corrected (It is 90% of the deaths caused by cervical cancer)

7. **Third paragraph reword ‘Breast and cervical cancers screening is a crucial strategy’ to ‘Breast and cervical cancer screening are crucial strategies….’**

Response: Corrected

8. **Line 6 – save lives not saves lives**

Response: Corrected

9. **Line6/7 ‘preserve quality of life’ not ‘preserves the quality of life’**

Response: corrected

10. **Para 4 last line: Reword ‘and can inform policymakers to assist in the development of policies and targeted interventions to improve screening uptake.’**

Response: corrected

11. **Second para line 2, consisting of 53**

Response: corrected

12. **Line 4 – ‘of whom’, not ‘of which’ (same word replacement needed, second last line of sampling section also)**

Response: corrected

13. **Line 5 typo delete 1 from 148.9 % male**

Response: Corrected

14. **Dependent variables: unclear – do these mean if the woman ever reported that a doctor or HCW had tested her? This is what I assume from the limitations section saying it was self-reported. Was there any explanation to participants about what being screened for either of these conditions would entail (eg breast examination or speculum use etc)? How do we know that the responses are valid/likely to indicate that screening occurred?**

Response: The dependent variable is based on the self-reporting by the women. Self-reporting is a limitation to this study which is included in the limitation section.

15. **What age groups are screen eligible in Nepal for each program? Noting that the sample comprises women aged 15-49 years and that the median age is 29, it is worth noting that the general start age for cervical screening per WHO guidelines is 30 and for breast screening in many countries it is 50. So, you would not expect most of these women to be screening even if they were aware of the programs and the programs were available. I suggest that the analysis should be redone or at least stratified by those who are age eligible for the programs rather than including younger women? You have documented in your results a very strong association between screening and age as would be expected for programs where younger people should not be screening.**

Response: We have redone the analysis. We have presented characteristics of different age group of women and later determine associations of CCS and BCS uptake among women aged 30 years and above.
